# Supplementary material for: Insulin-Like Growth Factor 2 mRNA-Binding Protein 1 (IGF2BP1) Is a Prognostic Biomarker and Associated with Chemotherapy Responsiveness in Colorectal Cancer
Source: Int J Mol Sci. 2021 Jun 28;22(13):6940. doi: 10.3390/ijms22136940 (PMC8267666; doi:10.3390/ijms22136940)
Supplement: Supplementary file 1 [file ijms-22-06940-s001.zip › ijms-1243527-supplementary.pdf]

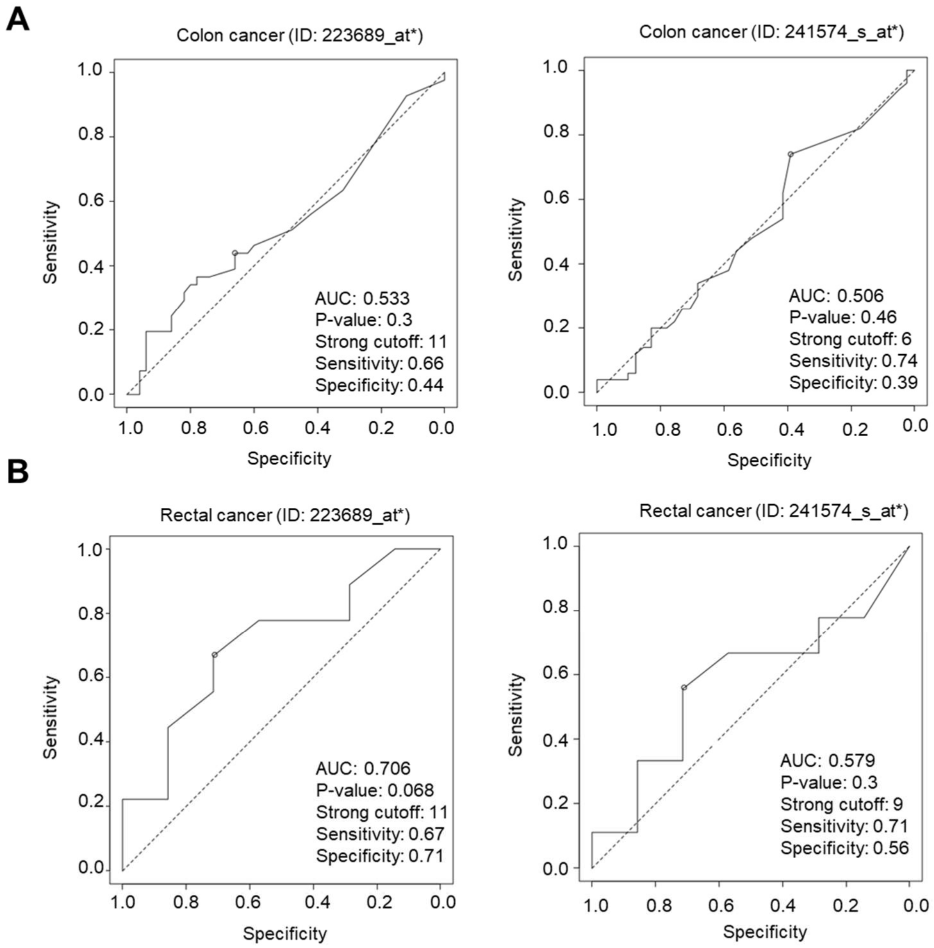

Supplementary figure 1: Examining roles of IGF2BP1 on the classification of responders and non-responders to chemotherapy in CRC patients. (**A**) The receiver operating characteristic (ROC) plot showed the sensitivity and specificity of IGF2BP1 expression (left panel: probe identify: 223689_at*; right panel: probe 241574_s_at*) for classifying chemotherapy responsiveness at the strong cutoffs in 91 colon cancer patients. AUC: area under curve. (**B**) The ROC plot showed the sensitivity and specificity of IGF2BP1 expression (left panel: probe identify: 223689_at*; right panel: probe 241574_s_at*) for classifying chemotherapy responsiveness at the strong cutoffs in 16 rectal cancer patients. AUC: area under curve.

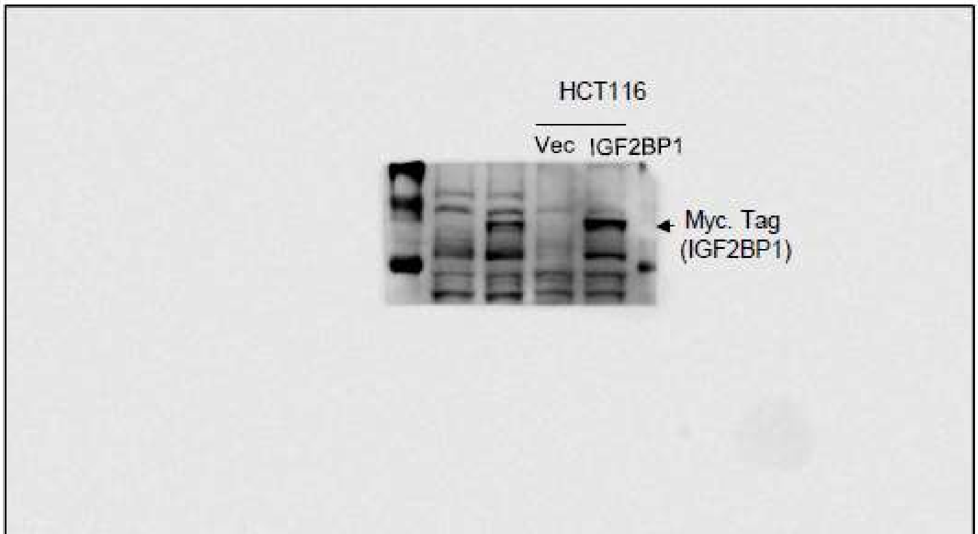

Figure 5a upper panel

File name: 20181127_myc-IGF2BP1-60sec222_6

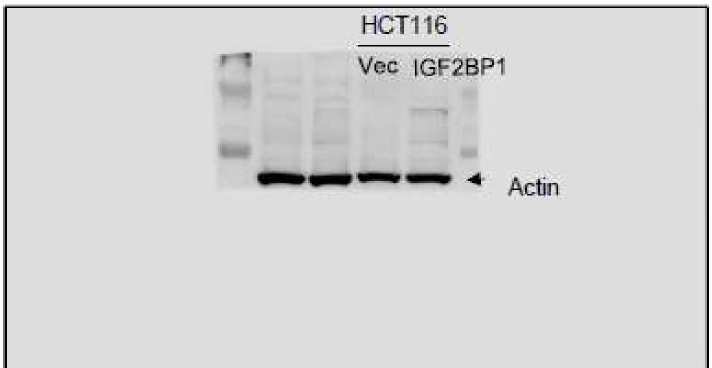

Figure 5a upper panel

File name: 20181128_actin-IGF2BP_222_2

Supplementary figure 2: The uncropped blots.
